# Analysis of rice ER-resident J-proteins reveals diversity and functional differentiation of the ER-resident Hsp70 system in plants

**DOI:** 10.1093/jxb/ert312

**Published:** 2013-10-23

**Authors:** Masaru Ohta, Yuhya Wakasa, Hideyuki Takahashi, Shimpei Hayashi, Kyoko Kudo, Fumio Takaiwa

**Affiliations:** Functional Transgenic Crops Research Unit, Genetically Modified Organism Research Center, National Institute of Agrobiological Sciences, Kannondai 2-1-2, Tsukuba, Ibaraki 305-8602, Japan

**Keywords:** BiP, endoplasmic reticulum, ER stress, Hsp70, J-protein, rice.

## Abstract

The heat shock protein 70 (Hsp70) chaperone system participates in protein folding and quality control of unfolded proteins. To examine the roles of co-chaperones in the rice Hsp70 chaperone system in the endoplasmic reticulum (ER), the functions of six ER-resident J-proteins (OsP58A, OsP58B, OsERdj2, OsERdj3A, OsERdj3B, and OsERdj7) in rice were investigated. The expression of *OsP58B*, *OsERdj3A*, and *OsERdj3B* was predominantly up-regulated in roots subjected to ER stress. This response was mediated by signalling through ATF6 orthologues such as OsbZIP39 and OsbZIP60, but not through the IRE1/OsbZIP50 pathway. A co-immunoprecipitation assay demonstrated that OsP58A, OsP58B, and OsERdj3B preferentially interact with the major OsBiP, OsBiP1, while OsERdj3A interacts preferentially with OsBiP5, suggesting that there are different affinities between OsBiPs and J-proteins. In the endosperm tissue, OsP58A, OsP58B, and OsERdj2 were mainly localized in the ER, whereas OsERdj2 was localized around the outer surfaces of ER-derived protein bodies (PB-Is). Furthermore, OsERdj3A was not expressed in wild-type seeds but was up-regulated in transgenic seeds accumulating human interleukin-7 (hIL-7). Since ERdj3A–green fluorescent protein (GFP) was also detected in vacuoles of callus cells under ER stress conditions, OsERdj3A is a *bona fide* vacuole-localized protein. OsP58A, OsP58B and OsERdj3A were differentially accumulated in transgenic plants expressing various recombinant proteins. These results reveal the functional diversity of the rice ER-resident Hsp70 system.

## Introduction

Plants provide an attractive production platform in terms of scalability, safety, and cost-effectiveness compared with conventional fermenting systems employing bacteria, yeast, and mammalian cells (Twyman *et al.*, 2003; Sharma and Shrama, 2009). Expressing recombinant proteins in plant seeds produces stable recombinant proteins with high yields (Takaiwa *et al.*, 2007). Since the seeds of cereal crops are staple foods, transgenic seeds containing pharmaceutical proteins enable the induction or suppression of the immune system through the oral administration of these edible vaccines (Takaiwa, 2011).

An expression system was previously developed in transgenic rice (*Oryza sativa*) seeds to produce recombinant bioactive, functional proteins (Takagi *et al.*, 2005; Yasuda *et al.*, 2006; Nochi *et al.*, 2007; Wakasa *et al.*, 2011*a*; Suzuki *et al.*, 2012; Yang *et al.*, 2012*a*, *b*). These recombinant proteins are linked to the N-terminal signal peptide and the C-terminal KDEL endoplasmic reticulum (ER) retention tag and then expressed under the control of an endosperm-specific promoter (Takaiwa *et al.*, 2007). When the recombinant proteins are accumulated at too high a level and/or have adverse effects on rice endosperm tissues, the transgenic seeds exhibit aberrant grain phenotypes, with floury, shrunken characteristics and decreased grain weights (Oono *et al.*, 2010; Wakasa *et al.*, 2012; Kudo *et al.*, 2013). In these seeds, the induction of a subset of genes associated with ER stress responses was observed, including genes encoding molecular chaperones, as well as ER-associated degradation (ERAD), which is part of the unfolded protein response (Oono *et al.*, 2010; Wakasa *et al.*, 2012; Kudo *et al.*, 2013). Since molecular chaperones are highly implicated in the refolding and removal of unfolded proteins, it is important to characterize molecular chaperones that function during ER stress responses to enable the stable accumulation of recombinant proteins in transgenic plants.

Molecular chaperones mediate the correct assembly of other proteins but are not themselves components of the final functional structures (Ellis, 1987). One of the chaperone systems, the heat shock protein 70 (Hsp70) system, consists of three types of chaperones, namely ATP-regulated Hsp70 (DnaK), the ATP-independent co-chaperone Hsp40 (DnaJ), and a nucleotide exchange factor (NEF). Hsp70 binds to non-native proteins as substrates to prevent their aggregation. The binding and release of the substrates are regulated by a cycle of ATP/ADP exchange. In this scenario, the interaction between substrates and Hsp70 is mediated by the J-domain of Hsp40 (Mayer *et al.*, 2000). Since Hsp40s also interact directly with unfolded peptides and can recruit Hsp70 to protein substrates (Young *et al.*, 2003; Young, 2010), Hsp40s play pivotal roles in substrate recognition and delivery to Hsp70 at the early stages of chaperone-mediated protein folding.

Hsp40, a J-domain-containing protein (J-protein), contains a conserved J-domain with a signature length of ~70 amino acids. J-proteins are involved in various cellular processes including *de novo* protein folding, translocation of polypeptides across cellular membranes, and degradation of misfolded proteins (Kampinga and Craig *et al.*, 2010). During the Hsp70 system-mediated protein folding processes, J-proteins initially bind to an unfolded client protein and deliver the client protein to Hsp70. Then, the J-proteins stimulate the ATPase activity of Hsp70, which is responsible for stabilizing the interaction of Hsp70 with client proteins. The stimulation of ATPase activity is achieved by specific interactions with the conserved tripeptide comprising histidine, proline, and aspartate (HPD) within the J-domain. This HPD motif is crucial for J-domain function, and point mutations in the HPD motif of DnaJ and DnaJ homologues result in the loss of the ability of J-proteins to interact with Hsp70 proteins (Feldheim *et al.*, 1992; Wild *et al.*, 1992; Wall *et al.*, 1995). J-proteins are ubiquitously localized in various organelles including mitochondria and the ER, as well as the cytosol. For example, there are 41 J-proteins in mammals; four of these are localized in mitochondria, six are ER-resident J-proteins, and the others are cytosolic (Kampinga and Craig *et al*., 2010). In *Arabidopsis*, sequence analysis revealed that there are ~90 genes encoding proteins with J-domains (Miernyk, 2001). *Arabidopsis* has six ER-resident J-proteins, five of which are structurally conserved among yeast, animals, and plants (Yamamoto *et al.*, 2008).

Immunoglobulin-binding protein (BiP) is an ER-resident Hsp70 with a signal peptide and an ER retention signal at its N- and C-termini. At least five *BiP* genes were previously identified in the rice genome. The expression of these genes is up-regulated by ER stress (Oono *et al.*, 2010; Wakasa *et al.*, 2011*b*; Hayashi *et al.*, 2012). It was previously shown that overexpression and knock-down of *OsBiP1* in rice seeds induces a severe ER stress response, resulting in a deterioration of grain properties (Yasuda *et al.*, 2009; Wakasa *et al.*, 2011*b*). Although plants such as rice, *Arabidopsis*, tobacco, and maize have multiple copies of *BiP* genes (Denecke *et al.*, 1991; Wrobel *et al.*, 1997; Noh *et al.*, 2003; Hayashi *et al.*, 2012), human and rodent BiP (*GRP97*) and yeast BiP (*Kar2*) are encoded by a single gene (Kampinga and Craig *et al*., 2010). It remains unknown why plants have multiple BiPs and whether these BiPs may play differential roles in the Hsp70 chaperone system.

To understand the ER-resident Hsp70 chaperone system, it is important to elucidate the networks that connect BiPs and J-proteins. In this study, six ER-resident J-proteins (encoded by *OsP58A*, *OsP58B*, *OsERdj2*, *OsERdj3A*, *OsERdj3B*, and *OsERdj7*) were identified and characterized in rice. Using a co-immunoprecipitation (Co-IP) assay, the differences in affinity between these ER-resident J-proteins and OsBiPs were demonstrated. These results reveal the functional diversity of the rice ER-resident Hsp70 system.

## Materials and methods

### Construction of plasmids

The Ubip-GFP-GluBter vectors were constructed by PCR using the set of primers listed in Supplementary Table S1 available at *JXB* online. To construct ER-resident J-protein–green fluorescent protein (GFP) fusion genes used in this study, the coding regions of the six ER-resident J-protein genes (*OsP58A*, *OsP58B*, *OsERdj2*, *OsERdj3A*, *OsERdj3B*, and *OsERdj7*) were amplified by PCR using the primers shown in Supplementary Table S1. The DNA fragments were inserted into the *Kpn*I sites of the Ubip-GFP-GluBter vector. The 3× FLAG tag sequence was amplified using the primers shown in Supplementary Table S1, and the DNA fragment was inserted into the *Kpn*I and *Sac*I sites of the 2×35S-Nos vector to produce the 2×35S-3× FLAG-Nos vector. To produce the J-protein-FLAG constructs, the coding regions of the six J-proteins were excised from their GFP fusion constructs by digestion with *Kpn*I and inserted into the *Kpn*I sites of the 2×35S-3×FLAG-Nos vector. To make the OsBiP–haemagglutinin (HA) fusion constructs, the coding regions of *OsBiP* genes (*OsBiP1*, *OsBiP2*, *OsBiP3*, and *OsBiP5*) were amplified by PCR with the primers listed in Supplementary Table S1. The DNA fragments were digested with *Bgl*II and inserted into the *Bam*HI sites of the Pro35S-ShΔ:2HA-6His vector (kindly provided by Dr Kagaya, Mie University). The orientation of the inserts was confirmed by PCR, followed by sequencing of the entire coding regions. The inserts in the sense orientation were used for further analysis.

### Transient expression of J-protein–GFP in rice protoplasts

The transient expression assay was carried out as described previously (Kawakatsu *et al.*, 2009). Protoplasts were prepared from cultured rice cells (Oc cells). Plasmid DNA for J-protein–GFP fusion constructs was introduced into protoplasts by electroporation. After overnight incubation at 28 °C, fluorescent images were obtained through a confocal laser-scanning microscope (FLUOVIEW FV10i-O; Olympus, Japan).

### Plant materials and growth conditions

*Oryza sativa* L. cv. Kita-ake plants were grown on 1/2 MS medium (1/2 Murashige and Skoog salt mix, 0.25% Gelrite, pH 5.7) at 25 °C under a 16h light/8h dark cycle. For stress treatments, 1-week-old seedlings were incubated in liquid MS medium containing 2mM dithiothreitol (DTT), 100mM NaCl, or 5 µg ml^–1^ tunicamycin (Tm).

### RNA extraction and reverse transcription–PCR (RT–PCR) analysis

Total RNA was extracted from roots using an RNeasy Plant Mini Kit (Qiagen, Germany). Total RNA was extracted from seeds as previously described (Takaiwa *et al.*, 1987). To remove genomic DNA, total RNA from seeds was treated with DNase I (TAKARA, Japan). First-strand cDNA was synthesized from 1 µg of total RNA using the SuperScript III First-Strand Synthesis System (Invitrogen, CA, USA) according to the manufacturer’s instructions. The cDNAs were amplified by PCR using Go-Taq polymerase (Promega, WI, USA) with gene-specific primers for *OsBiP1-5* and *17S rRNA* (Wakasa *et al.*, 2011*b*) and for the genes encoding J-proteins (listed in Supplementary Table S1 at *JXB* online).

### Co-immunoprecipitation analysis

Protoplasts from rice Oc cells were transfected with plasmid DNAs harbouring J-protein-FLAG and BiP-HA by electroporation and incubated overnight at 28 °C. Protoplasts were harvested by brief centrifugation and extracted with 200 µl of buffer containing 50mM TRIS-HCl pH 7.5, 150mM NaCl, 0.5% Triton X-100, and 1× Complete mini EDTA-free Protease Inhibitor Cocktail (Roche, Switzerland). After centrifugation at 12 000 *g* for 15min, the supernatant was mixed with anti-FLAG M2 Magnetic Beads (Sigma-Aldrich, MI, USA) for 2h at 4 °C to immunoprecipitate the J-protein-FLAG-tagged proteins. The beads were washed three times with NET buffer containing 50mM TRIS-HCl pH 7.5, 150mM NaCl, and 0.1% NP-40. The immunoprecipitated samples were eluted with 1× SDS loading buffer (50mM TRIS-HCl, pH 6.8, 2% SDS, 6% 2-mercaptoethanol, and 10% glycerol) and separated by 10% SDS–PAGE, followed by blotting onto PVDF membranes as described previously (Yamamoto *et al.*, 2006). Immunoblots were probed with anti-FLAG–horseradish peroxidase (HRP) or anti-HA–HRP (Sigma-Aldrich). The signals were detected using ImmunoStar (Wako, Japan) and ImageQuant LAS400 (GE Healthcare, Sweden). Signal intensities were analysed using ImageQuant TL software (GE Healthcare).

### Immunoblot analysis

Protein extraction from wild-type and transgenic rice seeds and immunoblot analysis were performed as described previously (Kudo *et al.*, 2013). Antibodies against OsP58A&B, OsERdj2, and OsERdj3A were prepared in this study. The NH_2_-AQEGQDNDPSTLFKR-OH, NH_2_-AHQKANSDIDSGSDD-OH, and NH_2_-NQAKKPAHTDQP KEN-OH peptides, which were derived from OsP58A&B, OsERdj2, and OsERdj3A, respectively, were synthesized and used to raise specific polyclonal antibodies (Scrum Inc., Japan). Since the peptide sequence of OsP58A&B described above is completely identical in OsP58A and OsP58B, these proteins could not be distinguished by the anti-OsP58 antibody. Polyclonal rabbit antibodies against human interleukin-7 (hIL-7) and cathelicidin were purchased from Sigma-Aldrich and Abcam (Cambridge, UK), respectively. Other rabbit polyclonal antibodies were prepared previously (Yasuda *et al.*, 2006; Oono *et al.*, 2010; Wakasa *et al.*, 2012).

### Confocal immunohistochemical analysis

Maturing seeds from wild-type and transgenic plants expressing hIL-7 were harvested at 18 days after flowering (DAF) and used for immunocytochemical analysis as described by Yasuda *et al.* (2006). Primary antibodies (anti-OsP58, anti-ERdj2, anti-ERdj3A, anti-OsBiP1, and anti-CNX) were used at a 1:500 dilution. The Alexa488-conjugated goat anti-rabbit IgG (Invitrogen) was used at a 1:500 dilution as a secondary antibody. Rhodamine B was used for staining of ER-derived protein bodies (PB-Is). For double staining, mouse anti-glutelin B (GluB) and rabbit anti-ERdj3A polyclonal antibodies were reacted simultaneously, followed by reaction with the Alexa488-conjugated goat anti-rabbit IgG and Alexa647-conjugated goat anti-mouse IgG (Invitrogen) at 1:500 dilutions as secondary antibodies. The samples were observed through a confocal laser-scanning microscope (FLUOVIEW FV10i-O; Olympus, Japan).

## Results

### Identification of ER-resident J-proteins from rice

Analysis of genome sequences revealed the presence of at least 104 putative J-protein genes in the rice genome (Sarkar *et al.*, 2013). Seven of these genes have putative signal sequences at their N-termini and exhibit high similarity to six ER-resident J-proteins from *Arabidopsis* (Yamamoto *et al.*, 2008). *Os07g0632600* encodes a polypeptide with 688 amino acid residues, which contains a region similar to the J-domain. However, this protein lacks HPD residues in the J-domain (see Supplementary Fig. S1 at *JXB* online). The HPD motif is necessary for interaction with Hsp70 proteins (Feldheim *et al.*, 1992; Wild *et al.*, 1992; Wall *et al.*, 1995). Thus, the possibility that *Os07g0632600* is an ER-resident J-protein was eliminated, and this protein was not subjected to further analysis.

*Os02g0195300* and *Os01g0977200* encode proteins with two regions similar to the tetratricopeptide repeat (TPR) followed by a J-domain, which are typical features of P58^IPK^ (Rutkowski *et al.*, 2007; Supplementary Fig. S2 at *JXB* online). The two genes were designated *OsP58A* and *OsP58B*, respectively. Os04g0307200 (OsERdj2) is an orthologue of AtERdj2A and AtERdj2B (Yamamoto *et al.*, 2008), but this protein is encoded by a single gene in the rice genome.

A full-length rice cDNA clone, AK063376, encodes a polypeptide with homology to AtERdj3A, but AK063376 lacks the first exon encoding the N-terminal portion including the J-domain. Thus, the missing first exon from genomic sequences was searched for and a new open reading frame (ORF) that includes the ATG initiation codon (Supplementary Fig. S3 at *JXB* online) was detected. *Os03g0293000* (*OsERdj3A*) encodes a polypeptide containing an N-terminal J-domain followed by a region with similarity to thioredoxin (Supplementary Fig. S2).

*Os05g0156500* (*OsERdj3B*) encodes a polypeptide with 347 amino acid residues, which contains an N-terminal J-domain, a zinc finger motif, and a DnaJ C-terminal domain (CTD; Supplementary Fig. S2 at *JXB* online).

When rice protein sequences with homology to the newly identified canine ERdj7 protein (Zahedi *et al.*, 2010) were searched for, Os12g0258200 produced the highest Blast score. Os12g0258200 is an orthologue of At1g61770, an *Arabidopsis* membrane-bound J-protein (Yamamoto *et al.*, 2008). Thus, Os12g0258200 was designated as OsERdj7 (Supplementary Fig. S2 at *JXB* online).

These results indicate that rice has a similar set of ER-resident J-proteins to *Arabidopsis*, but the number of each type of gene in rice is different from that in *Arabidopsis* (e.g. P58 and ERdj2).

### Subcellular localization of the rice ER-resident J-proteins

**Fig. 1.**
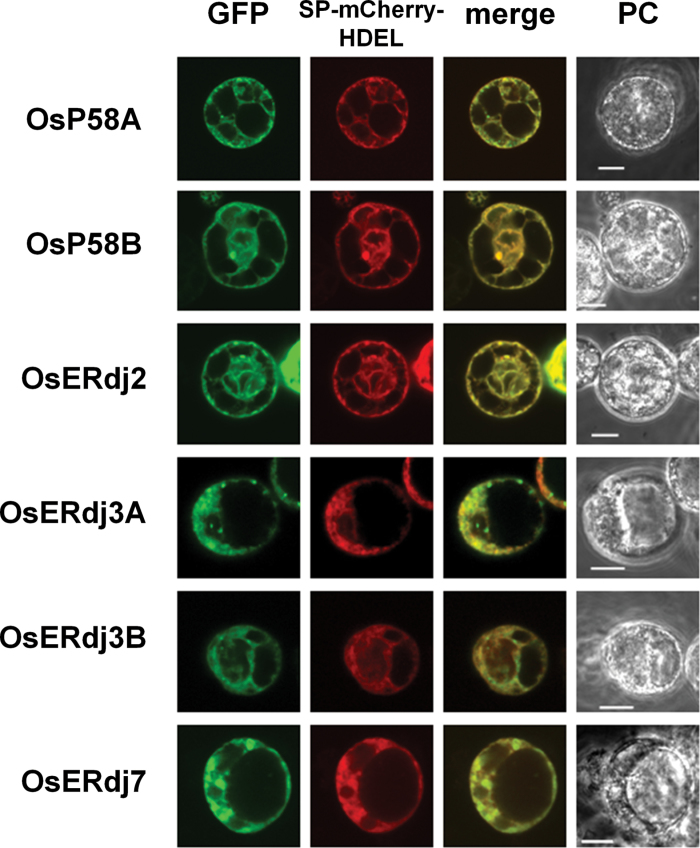
Subcellular localization of rice ER-resident J-proteins in protoplasts. An ER-localized mCherry (SP-mCherry-HDEL) fusion construct was transiently co-transfected with J-protein–GFP in rice protoplasts prepared from rice suspension-cultured cells (Oc cells). Cells were observed and photographed with a confocal laser-scanning (GFP and mCherry) and a phase contrast (PC) microscope. Bar=10 µm.

The subcellular localization of rice ERdjs was examined using protoplasts prepared from Oc cell suspension cultures that were transfected with DNA by electroporation. OsP58A, OsP58B, OsERdj2, OsERdj3A, OsERdj3B, and OsERdj7 were fused to GFP at their C-termini, and the J-protein–GFP fusion proteins were expressed in protoplasts. As shown in Fig. 1, the distribution of the six J-protein–GFPs coincided with an ER marker protein, sp-mCherry-HDEL, confirming the ER localization of these proteins. However, there was a slight difference in the distribution among the six J-protein–GFPs. In addition to the ER, OsERdj3A–GFP and OsERdj3B–GFP localized to punctae around the ER. To examine whether the OsERdj3A–GFP punctae co-localized with the OsERdj3B–GFP punctae, the protoplasts were co-transfected with OsERdj3B–mCherry fusion protein and OsERdj3A–GFP. OsERdj3B–mCherry punctae did not associate with the OsERdj3A–GFP punctae, indicating that these proteins formed different punctae (Supplementary Fig. S4 at *JXB* online). Small punctae of OsP58A–GFP were observed around the ER, but the sizes of the OsP58A–GFP punctae were smaller than those of OsERdj3A–GFP and OsERdj3B punctae (Fig. 1).

### Regulatory pathway for the expression of ER J-protein genes

**Fig. 2.**
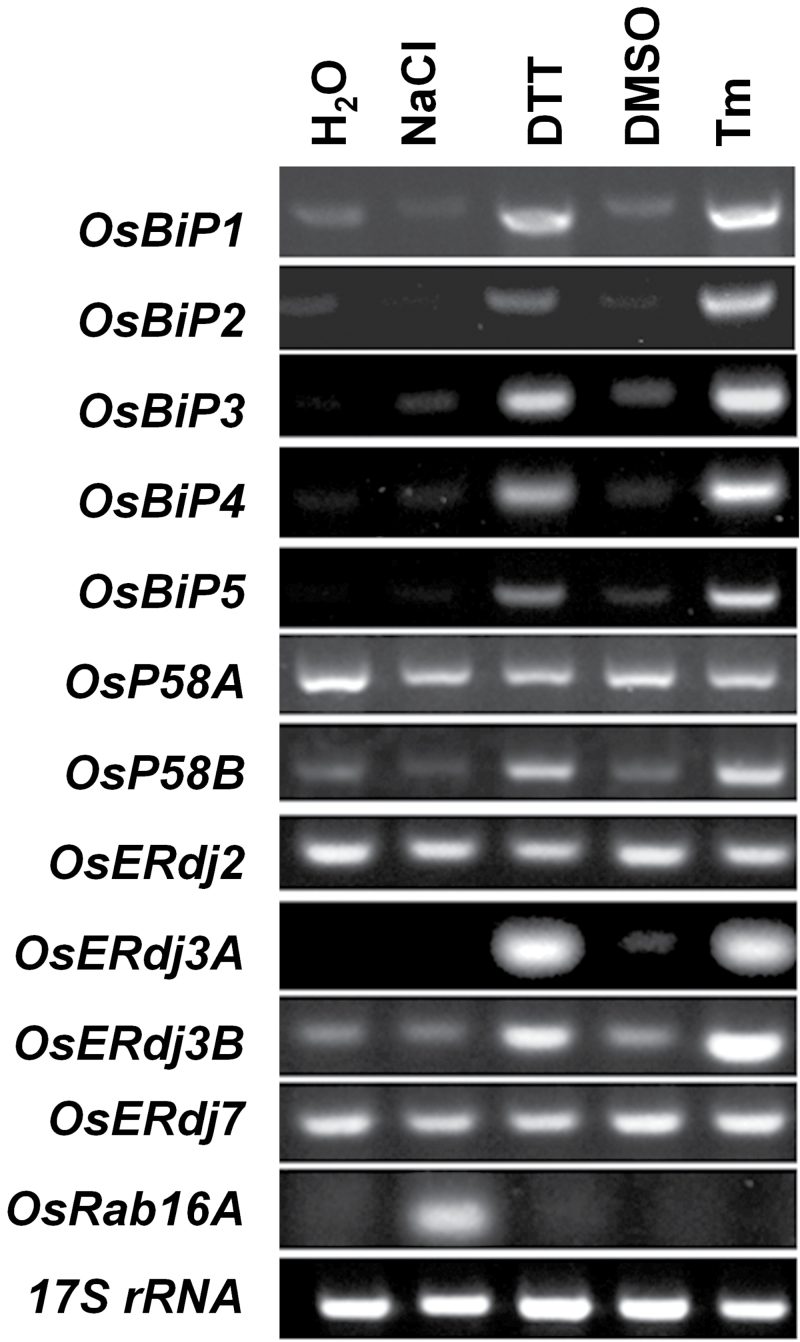
Expression profiles of genes encoding components of ER-resident Hsp70. Effects of various reagents that induce salt stress or ER stress responses. Total RNA was extracted from the roots of rice seedlings treated with 100mM NaCl, 2mM DTT, DMSO, or 5 µg ml^–1^ tunicamycin (Tm). Transcript expression levels were estimated by RT–PCR. *OsRab16A* was used as a positive control for a salt stress-responsive gene. *17S rRNA* was used as a loading control.

To investigate the regulatory pathway of the ER J-protein genes, RT–PCR analysis of individual ER J-protein genes was performed. The expression of *OsP58B*, *OsERdj3A*, and *OsERdj3B* was induced by DTT and Tm, but not by NaCl treatments in roots, indicating that *OsP58B*, *OsERdj3A*, and *OsERdj3B* are ER stress-responsive genes (Fig. 2). The expression pattern of these three genes was the same as that of *OsBiP1-5* (Fig. 2). In contrast, the expression of *OsP58A*, *OsERdj2*, and *OsERdj7* was not altered by DTT, Tm, or NaCl treatment, suggesting that the expression of these genes is not sensitive to ER stress (Fig. 2).

**Fig. 3.**
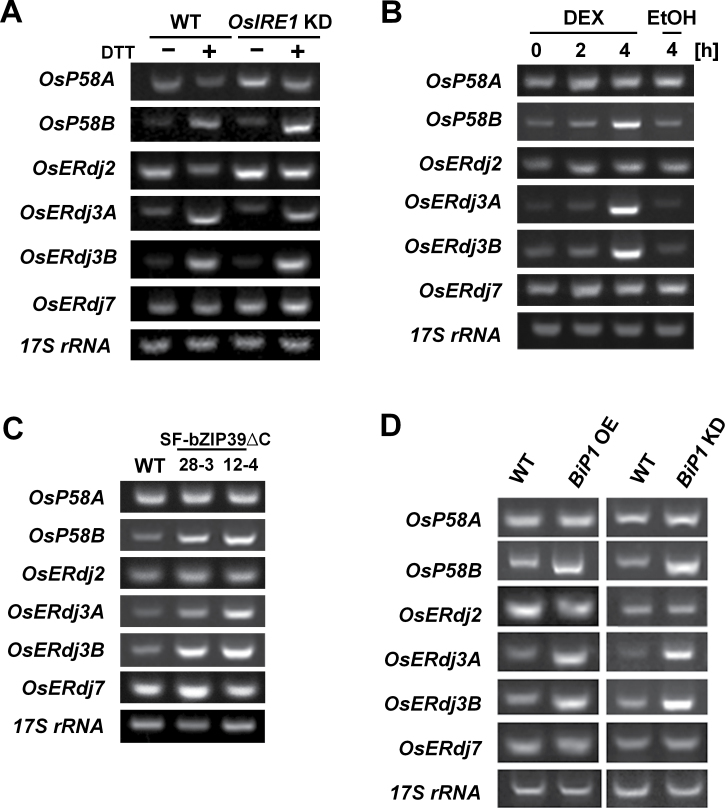
Regulation of ER J-protein gene expression. (A) Induction of ER J-protein genes in roots of wild-type (WT) and *OsIRE1* KD *Oryza sativa* (rice) lines by treatment with 2mM DTT. (B) Induction of ER J-protein genes by dexamethasone (DEX)-inducible::bZIP60ΔC in roots. Seedlings of transgenic plants (DEX-inducible::OsbZIP60ΔC) were treated with 30 µM DEX for the indicated period of time, and the roots were harvested for RNA isolation. For a control, DEX-inducible::OsbZIP60ΔC transgenic plants were treated with 0.01% (v/v) EtOH for 4h, and RNA was isolated from the roots. (C) Constitutive expression of *OsbZIP39ΔC* up-regulates the expression of *OsP58B*, *OsERdj3A*, and *OsERdj3B*. (D) Transcript levels of ER J-protein genes in ER-stressed seeds. The transcript levels were compared between seeds from WT and transgenic plants (*OsBiP1* overexpression; *OsBiP1* OE, *OsBiP1* knock-down; *OsBiP1* KD), which are known to elicit ER stress in seeds. Transcript levels were estimated by RT–PCR. *17S rRNA* was used as a loading control.

There are at least two main signalling pathways that mediate ER stress responses in plants. One pathway is composed of IRE1, the ER-resident receptor of ER stress, and a downstream transcription factor, OsbZIP50/AtbZIP60 (Deng *et al.*, 2011; Hayashi *et al.*, 2012). The induction of *OsP58B*, *OsERdj3A*, and *OsERdj3B* expression by DTT treatment was not affected in *IRE1* knock-down (*OsIRE1* KD) plants (Fig. 3A), indicating that these genes are regulated by an IRE1-independent pathway for ER stress responses. Another ER stress signalling pathway is mediated by the membrane-associated ATF6-like transcription factors OsbZIP39 and OsbZIP60 (Takahashi *et al.*, 2012; Hayashi *et al.*, 2013). When the truncated active form of OsbZIP60 without the transmembrane domain (TMD; OsbZIP60ΔC) was induced under the control of a dexamethasone (DEX)-inducible system, the expression of *OsP58B*, *OsERdj3A*, and *OsERdj3B* was activated (Fig. 3B). Notably, the transcript levels of *OsP58B*, *OsERdj3A*, and *OsERdj3B* also increased in transgenic rice that constitutively expressed a truncated active form of OsbZIP39 without the TMD (OsbZIP39ΔC) (Takahashi *et al.*, 2012) (Fig. 3C). In contrast, the transcript levels of *OsP58A* and *OsERdj7* were not affected by overexpression of OsbZIP39ΔC and OsbZIP60ΔC (Fig. 3B, C). These findings indicate that the expression of *OsP58B*, *OsERdj3A*, and *OsERdj3B* is mainly dependent on a signalling pathway that functions through the membrane-associated ATF6-like transcription factors OsbZIP39 and OsbZIP60.

The expression of genes encoding the ER-resident J-proteins was also examined in rice seeds. Seed-specific overexpression or RSIS (RNA silencing-inducible sequence)-mediated RNA silencing of *OsBiP1* resulted in the induction of ER stress in transgenic rice seeds, in which many ER stress-associated genes, including several *OsBiP* genes, were up-regulated (Wakasa *et al.*, 2011*b*). As shown in Fig. 3D, the transcript levels of ER stress-inducible ER J-protein genes such as *OsP58B*, *OsERdj3A*. and *OsERdj3B* were highly increased in *OsBiP1*-overexpressing (*OsBiP1* OE) and *OsBiP1* KD seeds. In contrast, the transcript levels of constitutively expressed ER J-protein genes such as *OsP58A*, *OsERdj2*, and *OsERdj7* were not affected in *OsBiP1* OE or *OsBiP1* KD seeds (Fig. 3D).

### Interaction of J-proteins with BiPs

**Fig. 4.**
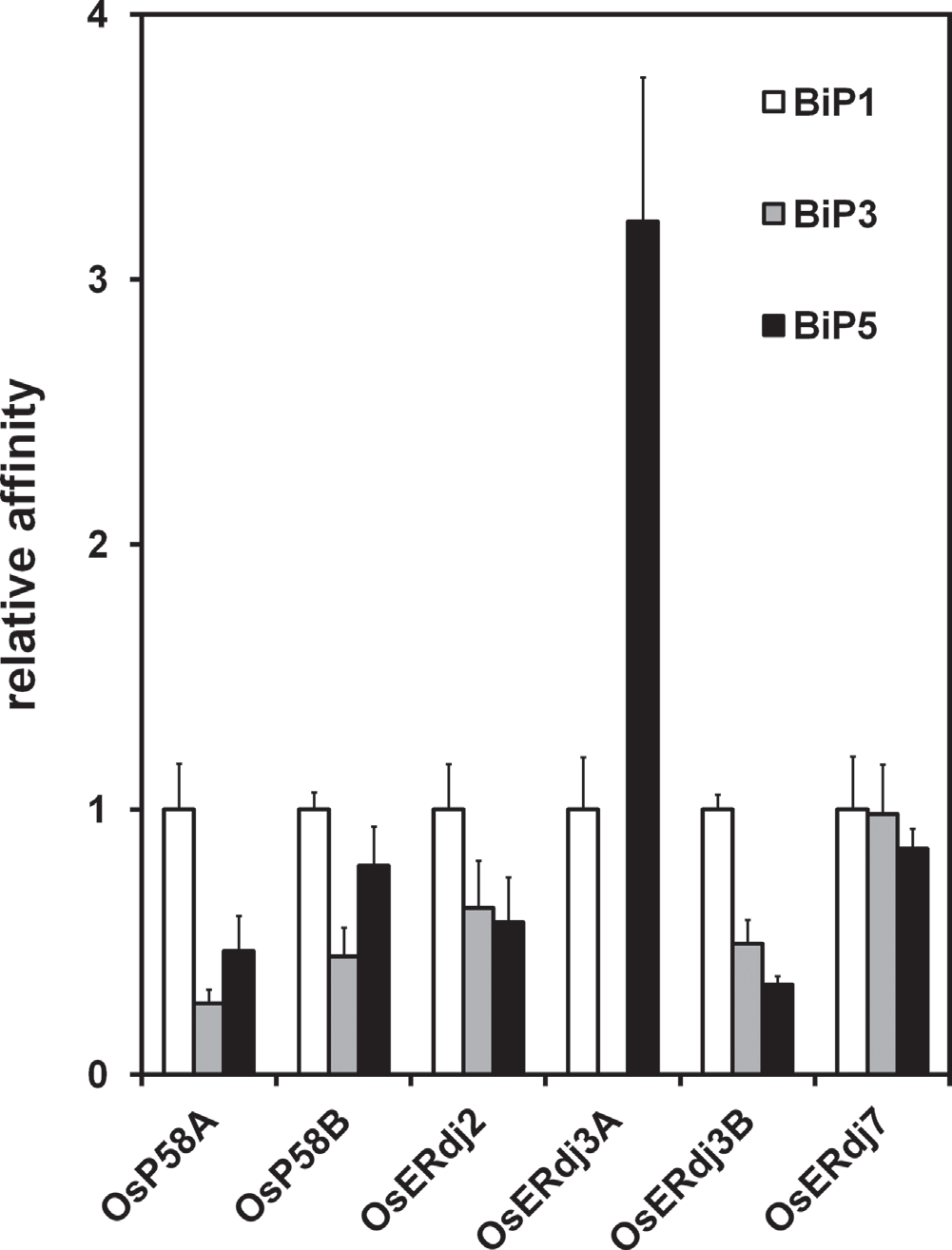
Interaction of ER J-proteins with BiPs in protoplasts. Plasmid DNA harbouring *OsBiP-HA* and rice *J-protein-3× FLAGs* were co-transfected into protoplasts prepared from rice suspension-cultured cells and incubated for 16h. Protein extracts from the protoplasts were subjected to immunoprecipitation using anti-FLAG. The immunoprecipitates were analysed by immunoblot analysis using anti-FLAG-tag and anti-HA-tag antibodies. Recovery levels of co-immunoprecipitated BiP-HAs were obtained by dividing the intensities of co-immunoprecipitated BiP-HAs by the intensities of the input BiP-HAs. Relative affinity represents the recovery of BiP-HAs relative to that of BiP1-HA immunoprecipitated by the same J-protein-FLAGs. Values represent the average of three independent experiments. Error bars represent the standard deviation (SD).

BiP is an ER-localized Hsp70 that receives substrate peptides from J-proteins and is activated by interactions with J-proteins (Kampinga and Craig, 2010). The interaction between the six rice ER-resident J-proteins and five OsBiPs was evaluated. Since the amino acid sequences of OsBiP4 and OsBiP5 are highly similar (Wakasa *et al.*, 2012), OsBiP5 was selected as a representative OsBiP. Full-length cDNAs for OsBiP1, OsBiP2, OsBiP3, and OsBiP5 were fused with double HA tags and a 6× His tag (2HA-6His) at their C-termini and these were expressed genes under the control of the enhanced *Cauliflower mosaic virus* (CaMV) 35S promoter carrying the first intron of Shrunken with an internal deletion (ShΔ) (Supplementary Fig. S5 at *JXB* online). Full-length rice J-proteins were fused with 3× FLAG tags at their C-termini and were expressed under the control of the double CaMV 35S promoter (Supplementary Fig. S5). To compare the binding affinities of the six J-proteins for different OsBiPs, it is important for the levels of the OsBiPs to be similar. The amount of plasmid DNA required to confer equal levels of individual OsBiP in protoplasts prepared from suspension-cultured rice Oc cells was first determined. OsBiP1-HA, OsBiP3-HA, and OsBiP5-HA accumulated to almost the same levels. However, OsBiP2 accumulated to a level of only 10% that of the other BiPs, even when larger amounts of plasmid DNA (20 µg) were used for transfection (Supplementary Fig. S6). Thus, OsBiP2-HA was not used in further experiments. The amount of plasmid DNA encoding the OsERdj-FLAGs was optimized for transfection. Except for OsERdj3A-FLAG, other J-protein-FLAGs accumulated at similar levels (Supplementary Fig. S6). When only OsBiPs were transfected into the protoplasts (mock), no OsBiPs were co-immunoprecipitated by anti-FLAG beads (Supplementary Fig. S7). However, when each J-protein-FLAG was co-transfected with different OsBiPs and the OsBiPs were co-immunoprecipitated using anti-FLAG beads, the amounts of co-immunoprecipitated OsBiP-HAs that were recovered differed among the J-protein-FLAGs (Supplementary Fig. S7). The recovery levels of co-immunoprecipitated OsBiP-HAs represent the relative affinities of individual J-protein-FLAGs for each OsBiP-HA. As shown in Fig, 4, there was little difference in the affinities of OsERdj2-FLAG and OsERdj7-FLAG for the three OsBiP-HAs. However, immunoprecipitation of OsP58A-FLAG, OsP58B-FLAG, OsERdj3A-FLAG, and OsERdj3B-FLAG showed that the affinities of the three OsBiP-HAs were different among these J-proteins. OsP58A-FLAG preferentially precipitated OsBiP1-HA over OsBiP3-HA (at a 4-fold higher affinity). OsBiP1-HA and OsBiP5-HA precipitated with OsP58B-FLAG to a similar extent. On the other hand, OsP58B-FLAG exhibited a lower ability to bind to OsBiP3-HA (*P* < 0.005, Fig. 4). OsERdj3B-FLAG preferentially precipitated OsBiP1-HA, with ~2.0- and 3.0-fold higher affinity than OsBiP3-HA and OsBiP5-HA, respectively (*P* < 0.005, Fig. 4). Although OsP58A, OsP58B, and OsERdj3B preferentially precipitated OsBiP1-HA, it is notable that OsERdj3A-FLAG preferentially precipitated OsBiP5-HA (at a level 4-fold higher than that of OsBiP1-HA; *P* < 0.005; Fig. 4). It was not possible to quantify the recoveries of OsBiP3-HA by OsERdj3A-FLAG due to the large degree of variation among different experiments. These results demonstrate that OsP58A, OsP58B, OsERdj3A, and OsERdj3B are functionally divergent, as they have different binding preferences for the three OsBiPs examined.

### Differential accumulation of J-proteins in transgenic seeds expressing various recombinant proteins during seed maturation

**Fig. 5.**
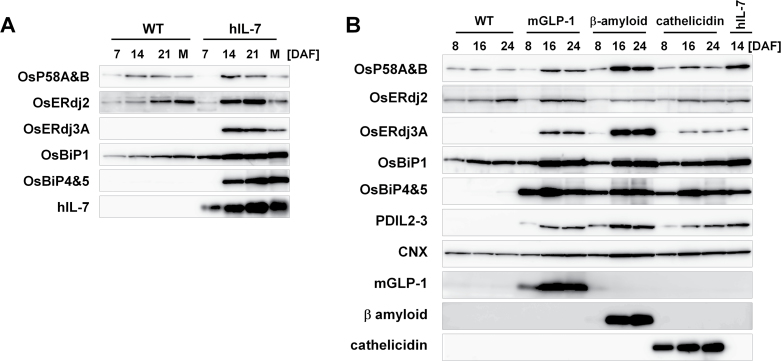
Accumulation of OsP58A&B, OsERdj2, and OsERdj3A in transgenic rice expressing various recombinant proteins. (A) Accumulation of ER J-protein genes in wild-type (WT) and human IL-7 (hIL-7)-expressing seeds during seed maturation. Total proteins were extracted from seed tissues at 7, 14, and 21 days after flowering (DAF) and from maturated seeds (M). (B) Accumulation of J-proteins in various transgenic seeds. Protein levels of OsP58A&B, OsERdj2, and OsERdj3A in WT and transgenic rice seeds expressing hIL-7, a 30 amino acid peptide hormone, mGPL-1, human β-amyloid, an antimicrobial peptide, and cathelicidin were estimated by immunoblot analysis.

The accumulation levels of ER-resident J-proteins during seed development were first examined by immunoblot analysis. As shown in Fig. 5A, the levels of OsP58A and OsP58B increased at 14 DAF and then decreased after 21 DAF. The level of OsERdj2 gradually increased over the course of seed maturation (Fig. 5A). In particular, the accumulation pattern of OsERdj2 was quite similar to that of OsBiP1. On the other hand, OsERdj3A was undetectable during seed development. Next, the expression levels of the J-proteins and chaperones were examined during seed development in transgenic rice seeds expressing hIL-7 driven by the glutelin *GluB-1* promoter (Kudo *et al.*, 2013). The accumulation level of hIL-7 gradually increases during seed development, beginning 7 DAF. In the hIL-7 seeds, OsBiP4 and OsBiP5 were induced beginning at 14 DAF, which is an indication of the ER stress response (Wakasa *et al.*, 2012). The level of OsBiP1 also increased in the hIL-7 seeds. OsERdj2 was detected from 7 DAF in both wild-type and hIL-7 seeds, and the level of this protein gradually increased during seed development in the wild-type. In contrast, the level of OsERdj2 reached a plateau at 21 DAF in the hIL-7 seeds and then decreased, although the overall accumulation levels were slightly higher in the in hIL-7 seeds than in the wild-type (Fig. 5A). OsERdj3A was accumulated along with the production of hIL-7 in seeds (Fig. 5A). These results suggest that the accumulation levels of OsP58A&B and OsERdj2 increase along with the increase in BiP accumulation during seed development, and the accumulation of OsERdj2 may be activated by ER stress induced by the accumulation of hIL-7. Furthermore, the finding that the expression of ERdj3A is induced by the accumulation of hIL-7 suggests that ERdj3A is related to ER stress responses such as ERAD.

It was previously reported that the levels of calnexin (CNX) and protein disulphide isomerase-like 2–3 (PDIL2-3) are differentially up-regulated in transgenic seeds accumulating various recombinant proteins and are altered according to properties of the recombinant proteins (Oono *et al.*, 2010). Therefore, whether the accumulation levels of J-proteins were also affected in transgenic seeds expressing various recombinant proteins including a modified version of glucagon-like peptide 1 (mGLP-1), β-amyloid (A β), and cathelicidin were examined (Yasuda *et al.*, 2005; Oono *et al.*, 2010; Wakasa *et al.*, 2012). As shown in Fig. 5B, the expression of chaperone proteins such as the ER stress marker proteins OsBiP4 and OsBiP5 (Wakasa *et al.*, 2012), as well as OsBiP1 and PDIL2-3, was induced by the accumulation of mGLP-1, A β, and cathelicidin in seeds. The expression levels of OsP58A, OsP58B, and OsERdj3A in the cathelicidin production line were lower than those in A β transgenic seeds at 16 DAF, although the expression levels of OsBiP1, OsBiP4, OsBiP5, and PDIL2-3 in A β were similar to those in the cathelicidin line at 16 DAF (Fig. 5B). The level of OsERdj3A in hIL-7 seeds at 14 DAF was similar to that in cathelicidin seeds from 16 to 24 DAF, but the levels of OsP58A and OsP58B were higher in hIL-7 seeds than in cathelicidin seeds (Fig. 5B). These results demonstrate that differential accumulation of OsP58A, OsP58B, and OsERdj3A was also observed in transgenic rice seeds expressing various recombinant proteins. In contrast, there was little difference in OsERdj2 levels in the seeds of the three transgenic plants (Fig. 5B).

### Intracellular distribution of J-protein in the endosperm of wild-type rice seeds

**Fig. 6.**
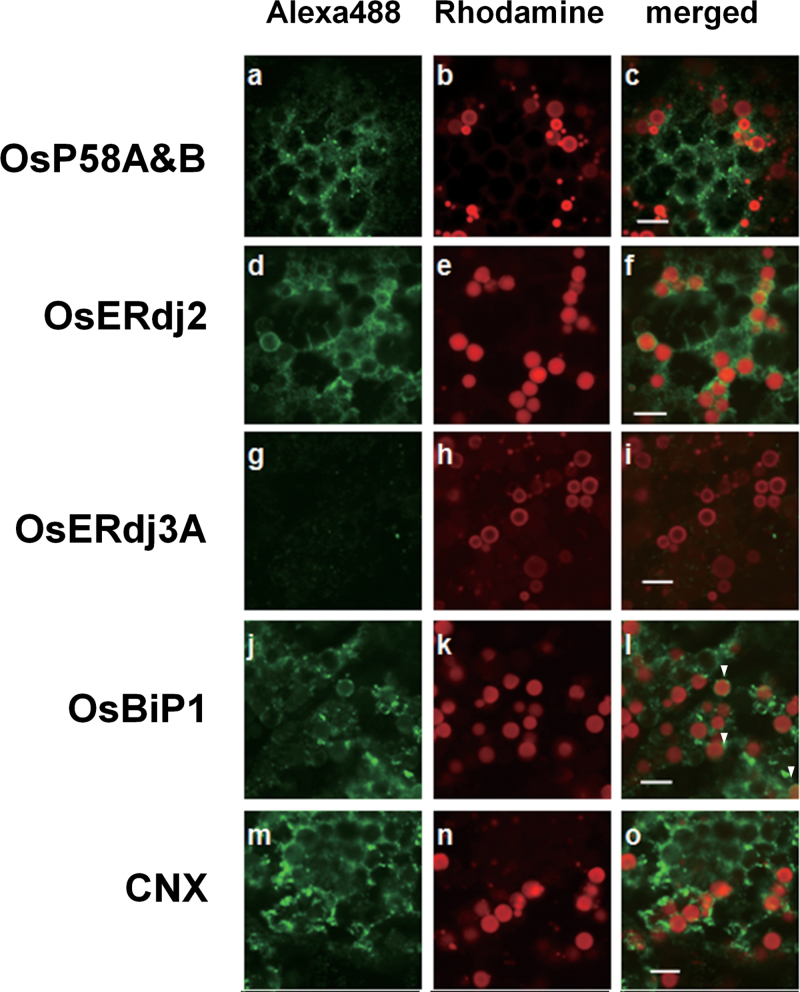
Subcellular distribution of OsP58A&B, OsERdj2, and OsERdj3A in wild-type seeds. Left panels show localization of OsP58A&B (a), OsERdj2 (d), OsERdj3A (g), OsBiP1 (j), and calnexin (CNX) (m); middle panels (b, e, h, k, and n) show localization of PB-Is (red); and right panels (c, f, i, l, and o) show the merged images of the left and middle panels. Arrowheads in l indicate PB-Is covered with OsBiP1 antibody. CNX was employed as a representative ER-marker protein. Bar=5 µm.

The endosperm of wild-type rice seeds contains two types of protein bodies, referred to as PB-Is and PB-IIs (Tanaka *et al.*, 1980; Krishan *et al.*, 1986). To examine the intracellular localization of J-proteins in endosperm cells, immunohistochemical analysis was performed with antibodies against rice OsP58A&B, OsERdj2, and OsERdj3A, as well as OsBiP1 using confocal immunofluorescence microscopy (Fig. 6). Furthermore, CNX protein was used as a marker protein for ER structure (Gupta and Tuteja, 2012). As shown in Fig. 6, OsP58A and OsP58B were localized mainly to the ER, which was similar to the localization of the ER-marker protein CNX (Fig. 6). Most of the OsERdj2 was also localized to the ER in wild-type seeds. In contrast to OsP58A and OsP58B, some OsERdj2 and OsBiP1 was also localized to the periphery of PB-Is (Fig. 6). Most of the PB-Is were covered with OsERdj2, while some PB-Is had OsBiP1 (shown in arrowheads). Since OsERdj3A was undetectable by immunoblot analysis in wild-type seeds (Fig. 5), the fluorescence pattern of OsERdj3A was not observed in wild-type seeds (Fig. 6).

### OsERdj3A is a vacuole-localized J-protein in rice

**Fig. 7.**
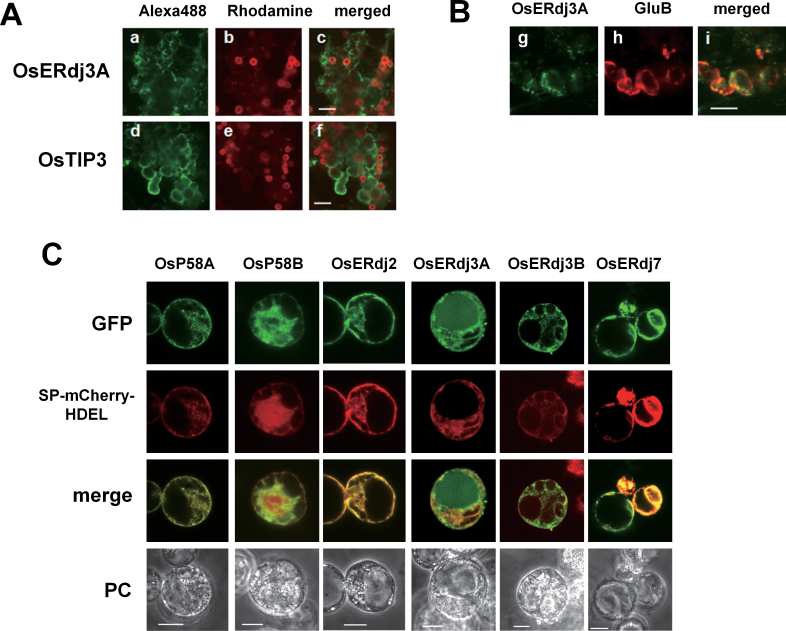
OsERdj3A localizes at PB-IIs in transgenic seeds expressing hIL-7. (A) Left panels show localization of ERdj3A (a) and OsTIP3 (d); middle panels (b and e) show localization of PB-Is (red); and right panels (c and f) show the merged images of left and middle panels (bar=10 µm). (B) (g, h, and i) Double staining images of hIL-7 seeds immunodecorated with anti-ERdj3A (g in green) and anti-glutelin B (GluB; h in red). (i) A merged image of g and h. Bar=5 µm. (C) OsERdj3A localizes to the vacuole in protoplasts under ER stress conditions. An ER-localized mCherry (SP-mCherry-HDEL) fusion construct was transiently co-transfected with J-protein–GFP in rice protoplasts. After 18h of incubation, the protoplasts were treated with 5 µg ml^–1^ tunicamycin for an additional 6h. Cells were observed and photographed with a confocal laser-scanning (GFP and mCherry) and a phase contrast (PC) microscope. Bar=10 µm.

To investigate the localization of OsERdj3A under ER stress conditions, maturing hIL-7 seeds were analysed by confocal immunofluorescence microscopy. The fluorescence pattern of OsERdj3A did not completely overlap with that of CNX (Figs 6m–o, 7A). Some OsERdj3A was observed as irregularly shaped vesicles that were similar to PB-IIs, as indicated by the fluorescence pattern of OsTIP3, which is a marker for PB-IIs (Fig. 7A). To confirm the localization of OsERdj3A in PB-IIs, double staining was performed using anti-ERdj3A and anti-GluB serum. As shown in Fig. 7B, the green fluorescence pattern of OsERdj3A overlapped with the red fluorescence pattern of GluB, indicating that OsERdj3A co-localized with GluB in PB-IIs. In contrast, the localization of OsP58A&B and OsERdj2 in hIL-7 seeds was quite similar to that in wild-type seeds, except for the reduction of OsERdj2 observed around the PB-I surface (Supplementary Fig. S8 at *JXB* online).

Since PB-IIs are derived from protein storage vauoles (PSVs), the localization of OsERdj3A in PB-IIs of hIL-7 seeds was not consistent with the ER localization of OsERdj3A observed in protoplasts (Fig. 1). One possible explanation for this result is that ER stress regulates the localization of OsERdj3A. To assess the possibility that ER stress alters the localization of OsERdj3A, protoplasts were treated with Tm after the transfection of OsERdj3A–GFP plasmid DNA. Under normal, unstressed conditions, most of the OsERdj3A–GFP was localized in the ER (Fig. 1). However, it is notable that the fluorescence pattern of OsERdj3A–GFP was instead observed in the vacuoles in response to Tm treatment (Fig. 7C). On the other hand, Tm treatment did not affect the localization of the other J-proteins (Fig. 7C). Taken together, these results suggest that OsERdj3A is a unique J-protein whose localization varies in response to ER stress.

## Discussion

In this study, six ER-resident J-proteins (OsP58A, OsP58B, OsERdj2, OsERdj3A, OsERdj3B, and OsERdj7) were identified and characterized in rice. A Co-IP assay demonstrated the differential affinity between individual OsP58A, OsP58B, OsERdj3A, and OsERdj3B proteins and cognate OsBiP1, OsBiP3, and OsBiP5. These results reveal the diversity of rice ER-resident J-proteins and the differential interactions of the ER-resident J-proteins with OsBiPs.

### Regulation of expression of genes encoding ER-resident J-proteins in rice

The accumulation of unfolded proteins in the ER in mammals results in the activation of three signalling pathways mediated by ATF6, IRE1, and PERK (Walter and Ron, 2010; Hetz, 2012). In plants, ER stress signalling pathways corresponding to the mammalian IRE1 and ATF6 pathways have been identified, but it is not clear whether the PERK pathway also operates in plants due to the lack of a PERK orthologue (for a review, see Iwata and Koizumi, 2012). In this study, it was demonstrated that IRE1 is not required for the induction of *OsP58B*, *OsERdj3A*, and *OsERdj3B* by ER stress. Instead, the overexpression of the ATF6-like transcription factor genes *OsbZIP39* and *OsbZIP60* resulted in the up-regulation of *OsP58B*, *OsERdj3A*, and *OsERdj3B* (Fig. 3). The induction of *OsBiP5* absolutely requires *OsIRE1/OsbZIP50*, whereas the induction of *OsBiP1* and *OsERdj3B* does not critically involve this signalling pathway (Hayashi *et al.*, 2012). Thus, ER stress regulates the expression of *OsP58B*, *OsERdj3A*, and *OsERdj3B* through an IRE1/OsbZIP50-independent pathway in rice. In mammals, the induction of *ERdj4* by ER stress requires XBP1 but not ATF6, while the induction of *P58* ^*IPK*^ and *ERdj3/HEDJ* by ER stress requires both XBP1 and ATF6 (Lee *et al.*, 2003; Wu *et al.*, 2007; Yamamoto *et al.*, 2007). These results imply that the regulatory mechanisms that induce ER-resident J-protein genes by ER stress in mammals are distinct from those in plants.

### Interaction of ER-resident J-proteins with OsBiPs

OsBiP1 was efficiently recovered by OsP58A, OsP58B, and OsERdj3B, while it was less efficiently recovered by OsERdj2, OsERdj3A, and OsERdj7 (Fig. 4). Under normal conditions, ER stress-associated OsBiP4 and OsBiP5 are not detected in seeds or roots (Wakasa *et al.*, 2012). Therefore, constitutively expressed J-proteins such as OsP58A, OsERdj2, and OsERdj7 might preferentially interact with OsBiP1 in rice. It is plausible that ER stress-associated BiPs might preferentially bind to the J-proteins induced by ER stress such as OsP58B, OsERdj3A, and OsERdj3B to form ER stress-specific J-protein–BiP complexes under ER stress conditions. Consistent with this notion, ER stress-inducible OsERdj3A has preferential affinity for OsBiP5 (Fig. 4). Thus, OsBiP5 and OsERdj3A probably form ER stress-specific J-protein–BiP complexes under ER stress conditions.

Another factor that affects the binding of BiPs to J-proteins is their subcellular localization. OsERdj2 is localized at the ER and the periphery of PB-Is in wild-type seeds, as is OsBiP1 (Fig. 6). Thus, OsERdj2 is likely to be involved in the formation of PB-Is, along with OsBiP1, in seed endosperm. The observation that OsERdj2 accumulates during seed maturation in wild-type seeds (Fig. 5A) suggests that OsERdj2 and OsBiP1 play specific roles in maturation of PB-Is. OsERdj2 is an integral membrane protein and an orthologue of yeast Sec63. Yeast Sec63 is involved in protein translocation across the ER and recruits Kar2 (BiP), thereby providing a driving force for translocation (Matlack *et al.*, 1999). Likewise, OsERdj2 and OsBiP1 may participate in the translocation of prolamins during seed maturation. On the other hand, the subcellular localization of OsP58A&B was more similar to that of CNX, OsBiP4, and OsBiP5 in ER-stressed endosperm tissue (Fig. 6). This finding suggests that OsP58B predominantly binds to OsBiP4&5 in response to ER stress, as OsP58B showed similar binding to OsBiP1 and OsBiP5, as shown in Fig. 4.

### OsERdj3A is a novel J-protein that is localized to the vacuole under ER stress

It should be noted that OsERdj3A is localized to the vacuole under ER stress conditions (Fig. 7). There are two possibilities explaining why OsERdj3A–GFP is not localized in vacuoles under normal conditions. One explanation is that the transport of OsERdj3A–GFP from the ER to the vacuole requires ER stress signalling. Since several genes encoding factors involved in protein trafficking are induced by ER stress (Urade, 2007; Wakasa *et al.*, 2011*b*), an ER stress-specific trafficking system might transport OsERdj3A–GFP to the vacuole. Another possibility is that OsERdj3A–GFP is constitutively transported to vacuoles and is rapidly degraded under normal conditions. In light of the finding that ER-resident proteins are constitutively transported to the vacuole for degradation (Tamura *et al.*, 2004), the latter possibility may be correct. Under ER stress conditions, a considerable amount of unfolded proteins is probably transported into vacuoles, which might competitively inhibit proteolytic degradation of OsERdj3A–GFP. Further experiments will be required to elucidate the transport mechanism for OsERdj3A–GFP to the vacuole.

Plant vacuoles play roles in proteolytic degradation as well as the storage of proteins (Paris *et al.*, 1996). OsERdj3A is localized to the protein storage type of vacuoles (PB-IIs) in rice seed endosperm tissues, since OsERdj3A co-localized with the storage protein glutelin (Fig. 7B). Notably, OsERdj3A was detected only under ER-stressed conditions (Figs 5, 7). OsERdj3A is likely to play a specific role in ER stress responses. In mammals, ER stress-inducible ER-resident J-proteins such as ERdj4 and ERdj5 are involved in the delivery of unfolded proteins to the ERAD machinery (Dong *et al.*, 2008; Ushioda *et al.*, 2008; Lai *et al.*, 2012). Like ERdj4 and ERdj5, OsERdj3A might play similar roles in ERAD. Whether the protein bodies where OsERdj3A is deposited have characteristics of lytic vacuoles remains to be determined. Compartments of lytic vacuoles are present inside the PSVs of tobacco and *Arabidopsis* seeds (Jiang *et al.*, 2001; Bolte *et al.*, 2011), raising the possibility that rice PSVs also play a role in proteolytic degradation.

It is not clear whether OsBiPs cooperate with OsERdj3A to perform functions in the vacuole. In the case of ERAD in mammalian cells, both ERdj4 and ERdj5 are associated with BiP (Dong *et al.*, 2008). Several lines of evidence support the vacuolar localization of BiP in plants. Subcellular fractionation analysis revealed ER-resident proteins such as PDI and BiP in the vacuolar fraction of cultured *Arabidopsis* cells (Tamura *et al.*, 2004). Furthermore, the export of BiP occurs via COPII-dependent transport to the Golgi apparatus, and BiP is proposed to play a role in quality control within the secretory pathway through vacuole disposal (Pimpl *et al.*, 2008). Deletion of HDEL from BiP results in the accumulation of BiPΔHDEL in lytic vacuoles (Pimpl *et al.*, 2008). Since OsERdj3A does not have a typical ER retention signal such as KDEL or HDEL, OsERdj3A might be detected in vacuoles more easily than OsBiPs. Co-IP experiments indicated that OsERdj3A preferentially interacts with OsBiP5 (Fig. 4). Thus, OsERdj3A and OsBiP5 are likely to be novel constituents of a quality control system within the secretory pathway destined for the vacuole disposal system.

### Characteristics of the ER-resident Hsp70 chaperone system in rice

In contrast to yeast and animals, plants have multiple BiPs; these BiPs are functionally distinct from each other. Large amounts of OsBiP1 are constitutively present in the ER, and knock-down of *OsBiP1* results in severe ER stress in endosperm tissue irrespective of the up-regulation of *OsBiP4&5* (Wakasa *et al.*, 2011*b*). These results suggest that the OsBiP1 plays major roles, and its function cannot be completely compensated for by OsBiP4&5. In this study, it was demonstrated that OsBiP1 is the most preferred partner for OsP58A, OsP58B, and OsERdj3B (Fig. 4). Since the rice ER-resident J-proteins examined in the current study can also interact with OsBiP3 and OsBiP5 (Fig. 4), various combinations of interactions between BiPs and J-proteins may occur depending on the ER stress conditions. This divergence of BiP–J-protein interactions may enable the fine-tuning of ER stress responses caused by different types of unfolded proteins, which would provide optimal ER stress responses suitable for an individual unfolded protein.

In this study, the diversity of rice ER-resident J-proteins was demonstrated. In contrast to OsBiP1 and OsBiP4&5, OsP58A&B and OsERdj3A were differentially accumulated in transgenic seeds expressing various types of recombinant proteins (Fig. 5B), raising the possibility that ER-resident J-protein levels are regulated in a more subtle manner than OsBiP levels in ER-stressed transgenic seeds. Therefore, manipulating genes encoding ER-resident J-proteins is likely to help improve the expression of recombinant proteins.

## Supplementary data

Supplementary data are available at *JXB* online.

Figure S1. Alignment of J-domain sequence of rice ER-resident J-proteins.

Figure S2. Schematic representation of ER-resident J-proteins from rice.

Figure S3. Gene structure of OsERdj3A.

Figure S4. OsERdj3A is not co-localized with OsERdj3B in rice protoplasts.

Figure S5. Schematic representation of the constructs used for co-immunoprecipitation (Co-IP) experiments.

Figure S6. Levels of BiPs and J-proteins.

Figure S7. Co-immunoprecipitation of OsBiP-HAs with J-protein-FLAGs.

Figure S8. Subcellular distribution of OsP58A&B and OsERdj2 in hIL-7 seeds.

Table S1. Primers used for plasmid construction and RT–PCR analysis.

Supplementary Data

## Abbreviations:

BiP: immunoglobulin-binding protein
Co-IP: co-immunoprecipitation
ER: endoplasmic reticulum
ERAD: ER-associated degradation
GFP: green fluorescent protein
Hsp70: heat shock protein
IL: interleukin
PB: protein body
