# Supplementary material for: Analysis of rice ER-resident J-proteins reveals diversity and functional differentiation of the ER-resident Hsp70 system in plants
Source: J Exp Bot. 2013 Oct 23;64(18):5429–41. doi: 10.1093/jxb/ert312 (PMC3871807; doi:10.1093/jxb/ert312)
Supplement: Supplementary Data [file supp_64_18_5429__index.html]

Analysis of rice ER-resident J-proteins reveals diversity and functional differentiation of the ER-resident Hsp70 system in plants — Analysis of rice ER-resident J-proteins reveals diversity and functional differentiation of the ER-resident Hsp70 system in plants — Supplementary Data 

# Analysis of rice ER-resident J-proteins reveals diversity and functional differentiation of the ER-resident Hsp70 system in plants

## Supplementary Data

Data files

**Files in this Data Supplement:**

- Supplementary Data - Supplementary Data
