# Supplementary material for: Analysis of rice ER-resident J-proteins reveals diversity and functional differentiation of the ER-resident Hsp70 system in plants
Source: J Exp Bot. 2013 Oct 23;64(18):5429–41. doi: 10.1093/jxb/ert312 (PMC3871807; doi:10.1093/jxb/ert312)
Supplement: Supplementary Data [file supp_ert312_jexbot102491_file001.pdf]

Supplemental data

Analysis of rice ER resident J-proteins has revealed diversity and functional differentiation of ER resident Hsp70 systems in plants

Masaru Ohta, Yuhya Wakasa, Hideyuki Takahashi, Shimpei Hayashi, Kyoko Kudo and Fumio Takaiwa*

***Corresponding author:** Fumio Takaiwa

```
                                   20                  40                  60
                                   |                   |                   |
        P58-1_JD DWYKILGISKTASAAETASAAEIKRAYKKLALQWHPDKNVDKREEAENMFRE-IAAAYEVLGDEDKRVRYD 70
        P58-2_JD ...............D.....D...................N..........-.................. 70
      OsERdj2_JD EP.S...LEPG..ESD------..KS.RR.SI.Y.....P.--P..HKY.V.F.SK..QA.T.PVS.EN.E 63
     OsERdj3A_JD .P..V..VD.S..QRD------.QK.FH..S.KY.....--.SKG.QEK.A.-.NN..DI.S..E..KN.. 62
     OsERdj3B_JD SY.DV.QVP.G..E-D-----Q...S.R....KY.....PNN-...NKR.A.-.NN...I.T.QE..KI.. 63
      OsERdj5_JD .C.DL..VKQD.NVS.------..K..Y..S.KH.....P.--P.SRKL.VK-..N...I.K..ST.GQ.. 62
 Os07g0632600_JD -H.YA...ERF.PVE------RVVE..EQ.SKE.LAET.QQTTVDIIK-----.RY...L.TNPILKRD.. 59
```

Fig. S1. Alignment of J domain sequence of rice ER resident J-proteins. Residues identical to OsP58A are shown in dot. A consensus sequence, HPD, is underlined.

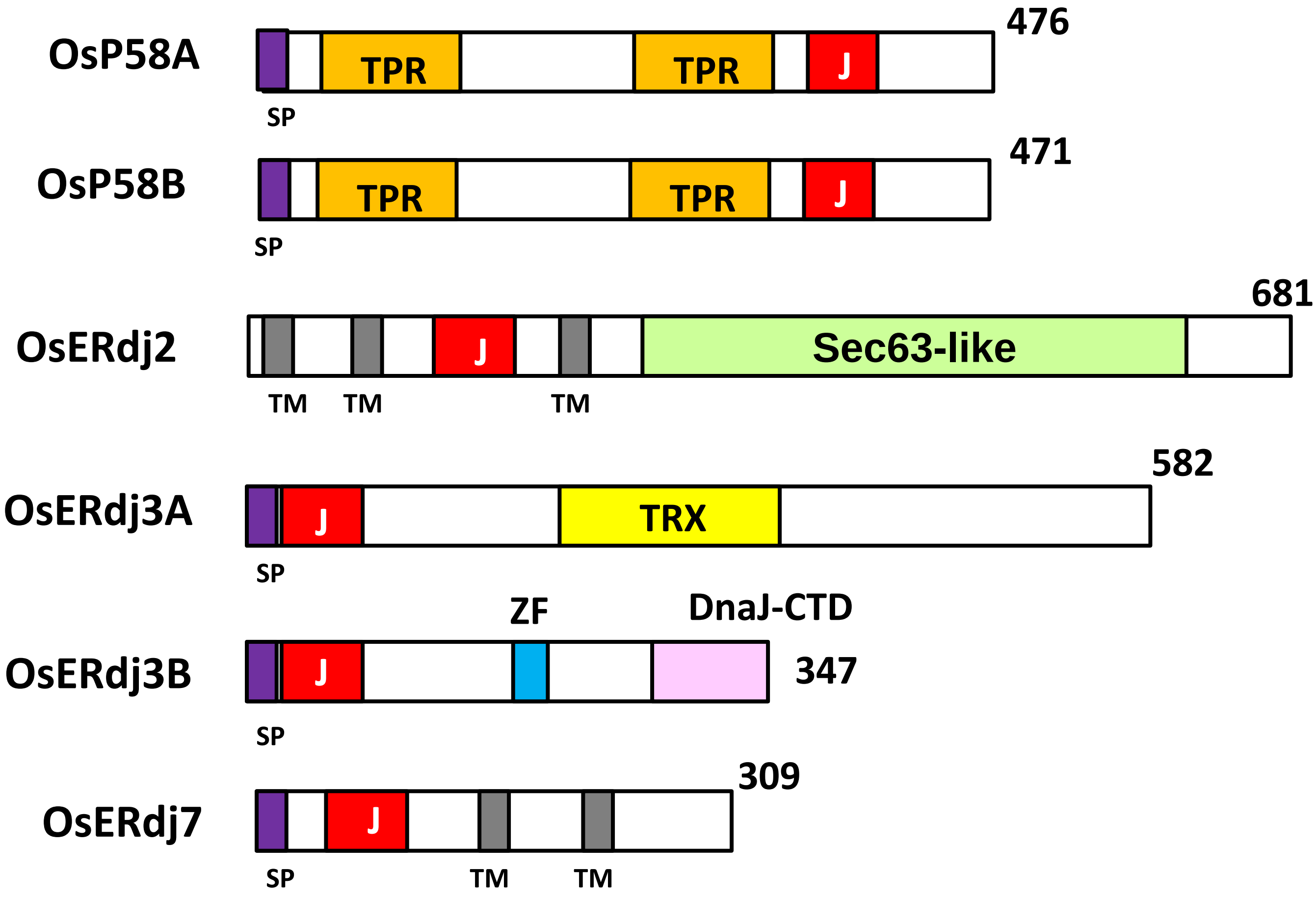

Fig. S2. Schematic representation of ER resident J-proteins from rice. Domain structures are shown. Red and purple boxes indicate J-domain and signal sequence, respectively. Gley box indicates transmembrane region (TM). Axially domains such as tetratricopeptide repeat (TPR), Sec63-like domain, thioredoxin domain (TRX), zinc finger motif (ZF), C-terminal domain of DnaJ (DnaJ-CTD) are indicated in each J-protein.

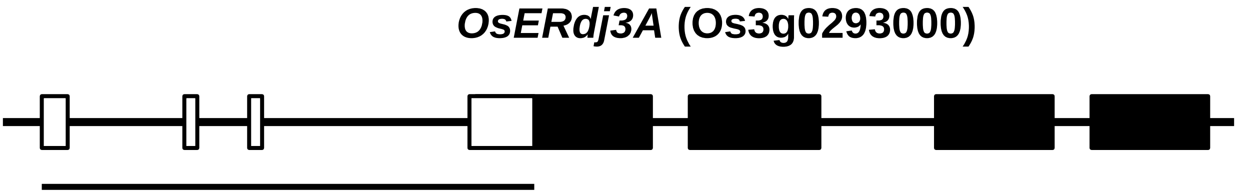

Fig. S3. Gene structure of OsERdj3A. Boxes represent exons. An underline represents the genomic region containing the missing exons. White box shows the new co ding sequences identified in this study.

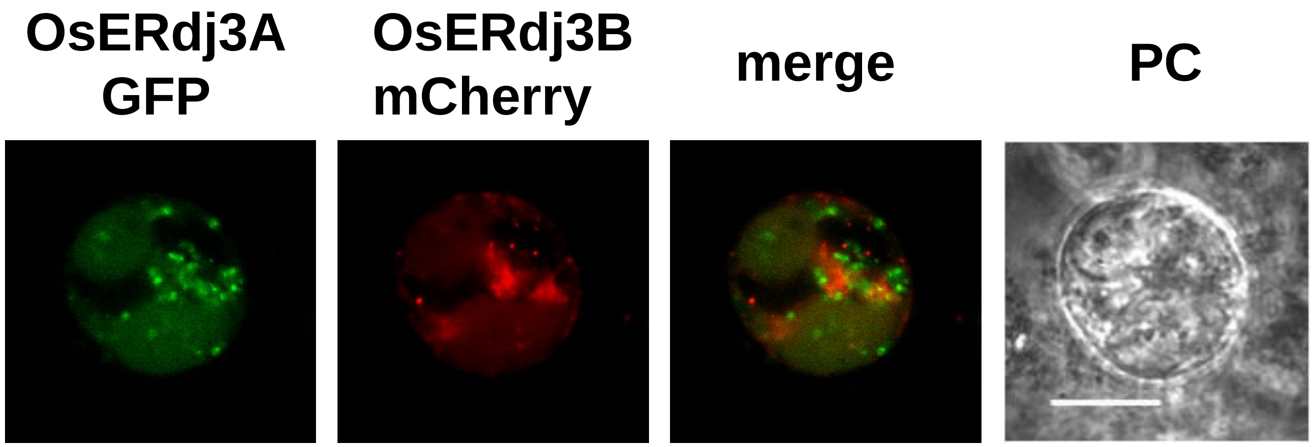

Fig. S4. OsERdj3A is not co-localized with OsERdj3B in rice protoplasts. OsERdj3A is not co-localized with OsERdj3B in rice protoplasts. OsERdj3B-mCherry fusion construct was co-transfected with OsERdj3A-GFP transiently in rice protoplasts prepared from rice suspension cultured cell, Oc cell. Cells were observed and photographed with a confocal laser-scanning microscope. Bar = 10 mm.

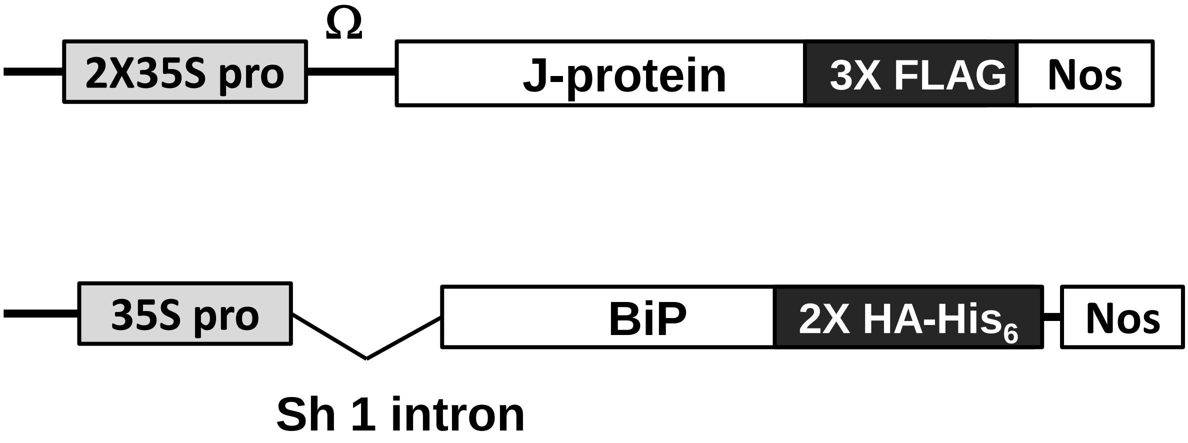

Fig. S5. Schematic representation of the constructs used for Co-immunoprecipitation (Co-IP) experiments. Double HA and His_6 tags were fused C-terminus to OsBiP and the resulting fusion constructs (BiP-HA) were expressed under control of CaMV 35S promoter (35S pro). J-protein-3x FLAG tag fusion genes were inserted between double 35S promoter (2X35S pro) and Nos terminator (Nos). Ω indicates a translational enhancer sequence derived from tobacco mosic virus.

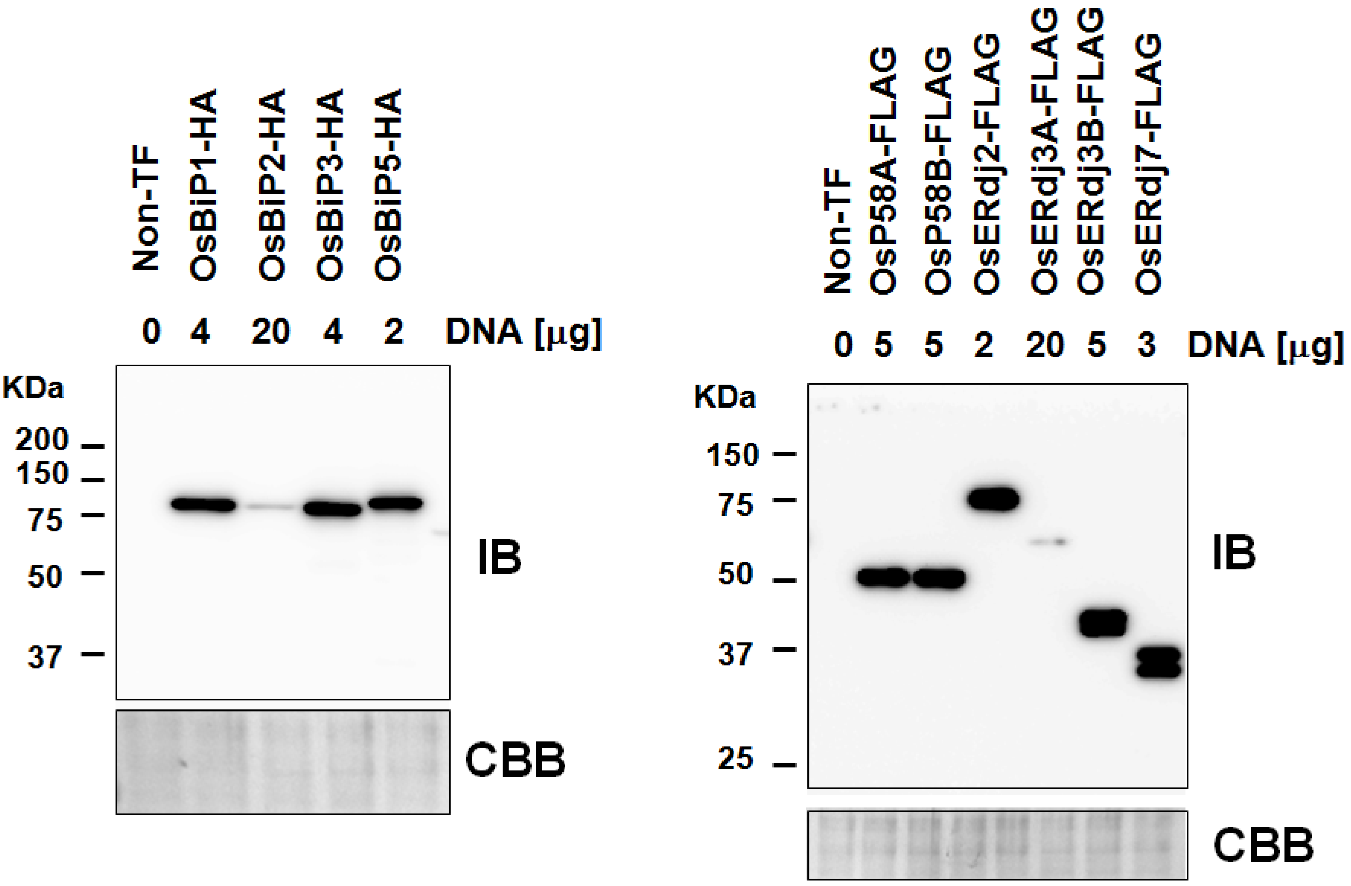

Fig. S6. Protein levels of BiPs (left) and J-proteins (right) in protoplasts. Plasmid DNA harbouring OsBiP-HA and the rice J-protein-3x FLAGs were co-transfected into protoplasts prepared from rice suspension-cultured cells and incubated for 16 h. The amount of the plasmid DNA (µg) was indicated top of the panels. Non-TF indicates a negative control of the transfection without plasmid DNA. IB indicates results of immunoblot analyses. BiPs and J-proteins were detected with monoclonal antibodies against FLAG-tag and HA-tag, respectively. CBB staining pattern was shown for loading control.

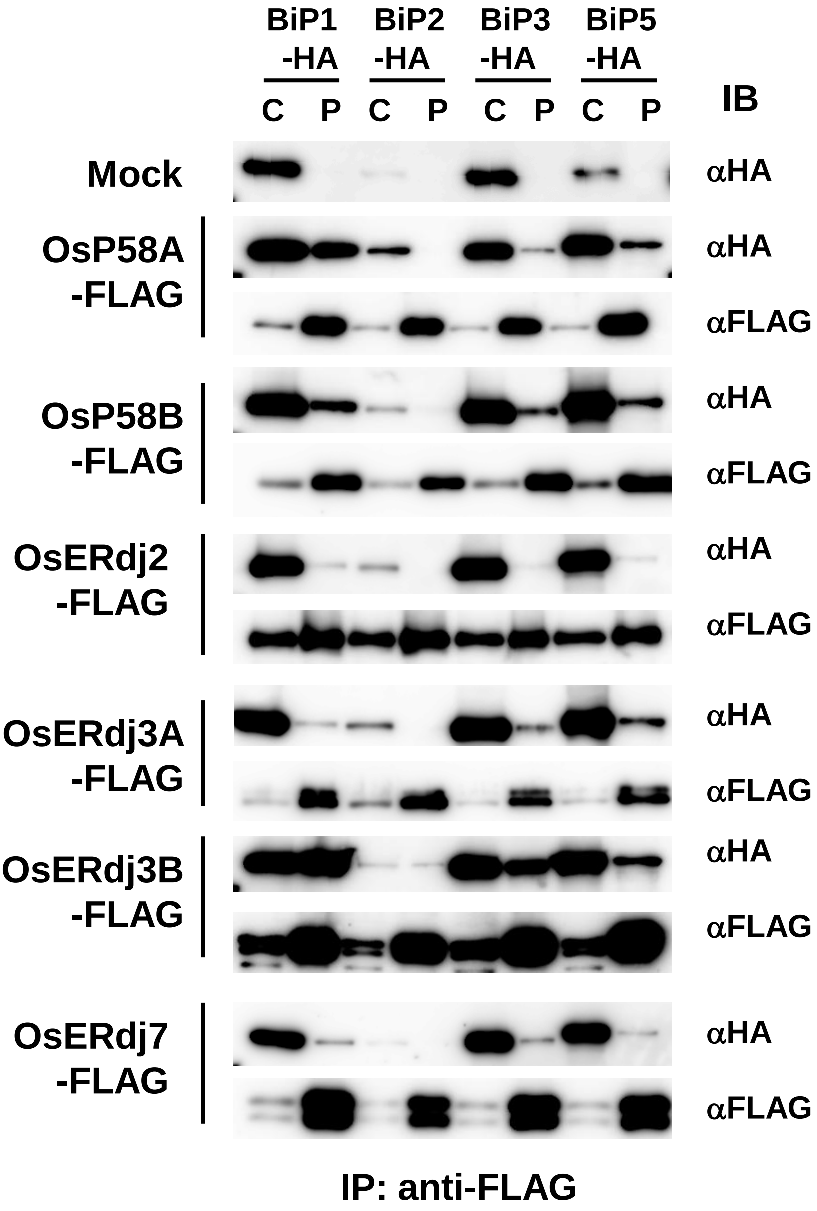

Fig. S7. Co-immunoprecipitation of Os-BiP-HAs with the J-protein-FLAG. Protein extracts from the protoplasts expressing OsBiP-HA and the rice J-protein-FLAG were subjected to immunoprecipitation using anti-FLAG. The immunoprecipitates were analyzed by immunoblot analysis using the anti-FLAG-tag and the anti-HA-tag antibodies. C represents 2% (v/v) of the starting crude lysate used for immunoprecipitation. P represents the imminoprecipitated proteins.

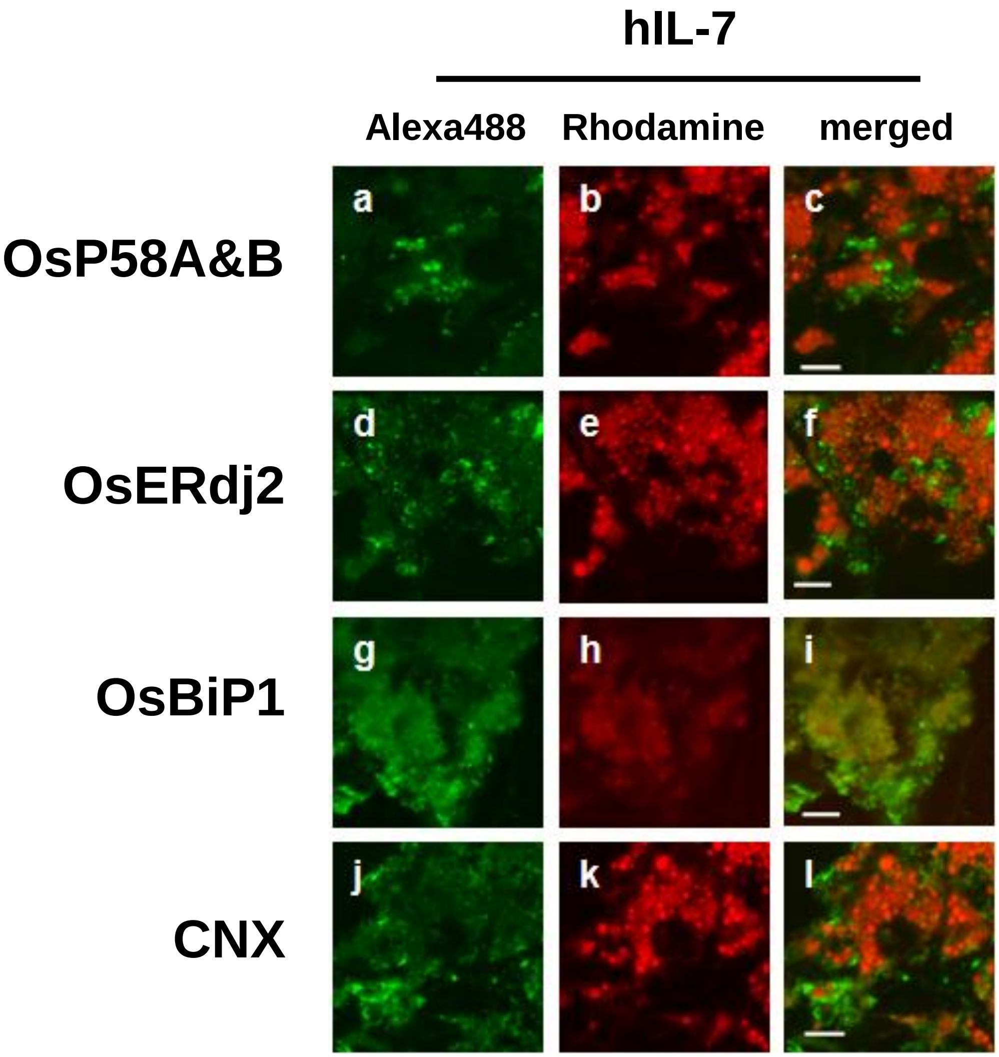

Fig. S8. Subcellular distribution of OsP58A&B, OsERdj2 in hIL-7 seeds. Left panels show localization of OsP58A&B (a), OsERdj2 (d), OsBiP1 (g) and calnexin (CNX) (j), middle panels (b, e, h and k) show localization of PB-I (red) and right panels (c, f, i and l) show the merged images of left and middle panels. Bar = 5 mm.

Table S1. Primers used for plasmid construction and RT-PCR analysis

Plasmid construction

Construct name	primer	sequence
Ubip-GFP-GluBter	GFP-3	5'-TGGGTACCATGGTGAGCAAGGGCGAGGAG-3'
	GFP-4	5'-CGAGAGCTCTTACTTGTACAGCTCGTCCATG-3'
OsP58A-GFP	P58-1-3	5'-GATGGTACCATGGTGGCGATGGCGCGGTG-3'
	P58-1-2	5'-CCGGTACCTCCAAAGTTGAACTGAAAG-3'
OsP58B-GFP	P58-2-3	5'-GATGGTACCATGGCGCGGTGGCCGTGGAG-3'
	P58-2-2	5'-CCGGTACCTCCAAAGTTGAACTGAAATC-3'
OsERdj2-GFP	ERdj2-12	5'-GTGGTTTAAGGTACCATGGCGGCGG-3'
	Erdj2-10	5'-AGGTACTATAGGTACCATCACT-3'
OsERdj3A-GFP	ERdj3A-11	5'-TAAGGTACCATGGGAATCCCTGTCCGATCTC-3'
	ERdj3A-10	5'-CGGCTGGTACCAAGAACTGGTTTTCGTCCGGC-3'
OsERdj3B-GFP	ERdj3B-9	5'-GGATGGTACCATGGCGGCGCCGCGGTGGATAG-3'
	ERdj3B-8	5'-TCGGGGTACCGAGAATGCTCTTCAGCTTGGAC-3'
OsERdj7-GFP	ERdj5-7	5'-ATGCCCCGGGATGTCCCAAGTTGGATCCGCCG-3'
	ERdj5-8	5'-ACAGGGTACCTCTACGCTTTGATTCCTTTCTC-3'
2x35S-3xFLAG-Nos	FLAG-1	5'-AAAGGTACCATGGCTTCCTCCGATTACAAGG-3'
	FLAG-2	5'-TTGTACAAGAGAGCTCGGTTTACTTATCATC-3'
OsBiP1-HA	BiP1-3	5'-AATCAGATCTATGGATCGGGTTCGCGGATGC-3'
	BiP1-4	5'-GCATAGATCTCAGCTCGTCATGCTCGTCG-3'
OsBiP2-HA	BiP2-5	5'-AATCAGATCTATGGCGAGAGATAAGCAATCTG-3'
	BiP2-4	5'-TCTAAGATCTAGCTCATTCACGTCGTCG-3'
OsBiP3-HA	BiP3-5	5'-AATCAGATCTATGGCGCGCGGCGCAACGTGGA-3'
	BiP3-4	5'-CATTAGATCTAAGCTCATCGTGATCGTCG-3'
OsBiP5-HA	BiP5-6	5'-GACGAGATCTATGGCGCGGCCGCGGCGCGCG-3'
	BiP5-2	5'-GCATAGATCTAAGCTCATCGTGGTCATCCTC-3'

RT-PCR

Gene name	primer	sequence
OsP58A	P58-1-4	5'-TCAGGATCTAAAAGAGGCTG-3'
	P58-1-5	5'-AATGGAATGTGTATTGCTGG-3'
OsP58B	P58-2-4	5'-TCAGGATCTAAAAGAAGCTT-3'
	P58-2-5	5'-AATGGAATGTGTATTGCTGA-3'
OsERdj2	ERdj2-1	5'-ATGACGAGGAGGATGAGAAGAG-3'
	ERdj2-2	5'-TGCACAAAGGGCAAAAACAACG-3'
OsERdj3A	ERdj3A-7	5'-AAGGTTCGTGGTTTCTGTTTTG-3'
	ERdj3A-8	5'-TAAGGAGGTACTACACTATAAG-3'
OsERdj3B	ERdj3B-5	5'-AGGTGGGAAGGAAAACTGCCTC-3'
	ERdj3B-6	5'-ATGACATGAGAAACAAAATAAG-3'
OsERdj7	ERdj5-1	5'-AGGTGCTCAGTTGGGAATTTTG-3'
	ERdj5-2	5'-TTCCCATGTATTAGCAGGTATC-3'
